# Supplementary material for: Characterization and Modification of Red Mud and Ferrosilicomanganese Fines and Their Application in the Synthesis of Hybrid Hydrogels
Source: Polymers (Basel). 2022 Oct 14;14(20):4330. doi: 10.3390/polym14204330 (PMC9608683; doi:10.3390/polym14204330)
Supplement: Supplementary file 1 [file polymers-14-04330-s001.zip › polymers-1900479-supplementary.pdf]

In order to study the dispersion of the inorganic phases in the polyacrylamide matrix, TEM micrographs were taken of the hybrid hydrogels at low (0.1% by mass) and high (10% by mass) concentration of the inorganic phases. The results show for both clays (red mud and ferrosilicomanganese fines) that the increases in the concentration of the inorganic phases tend to generate agglomerates of particles that form heterogeneities in the material (Figure S1b and S1d).

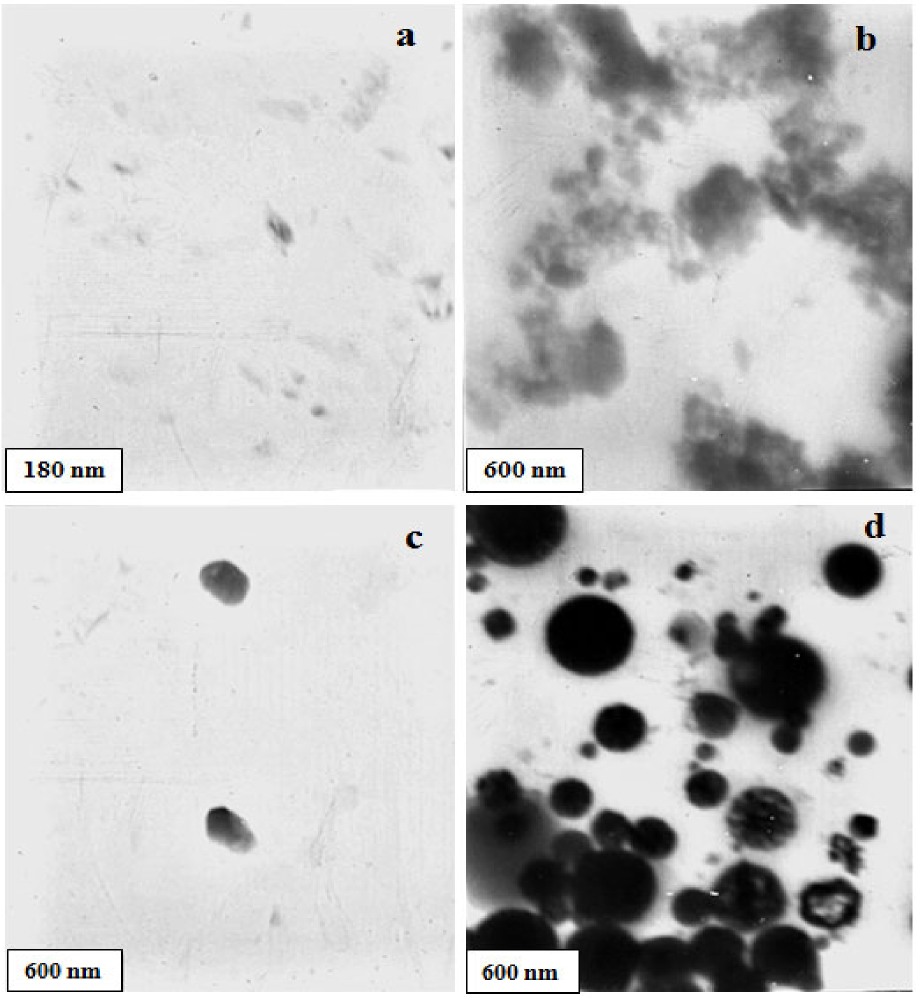

Figure S1. TEM micrograph for hybrid polyacrylamide/RM hydrogels: a) %RM=0.1%; b) %RM=10%) and polyacrylamide/FeSiMn: c) %FeSiMn=0.1%; d) %FeSiMn=10%. .
